# Comparative visualization of protein secondary structures

**DOI:** 10.1186/s12859-016-1449-z

**Published:** 2017-02-15

**Authors:** Lucia Kocincová, Miroslava Jarešová, Jan Byška, Július Parulek, Helwig Hauser, Barbora Kozlíková

**Affiliations:** 1 Masaryk University, Brno, Czech Republic; 2 University of Bergen, Bergen, Norway

**Keywords:** Molecular visualization, Molecular sequence analysis, Molecular structure and function

## Abstract

**Background:**

Protein function is determined by many factors, namely by its constitution, spatial arrangement, and dynamic behavior. Studying these factors helps the biochemists and biologists to better understand the protein behavior and to design proteins with modified properties. One of the most common approaches to these studies is to compare the protein structure with other molecules and to reveal similarities and differences in their polypeptide chains.

**Results:**

We support the comparison process by proposing a new visualization technique that bridges the gap between traditionally used 1D and 3D representations. By introducing the information about mutual positions of protein chains into the 1D sequential representation the users are able to observe the spatial differences between the proteins without any occlusion commonly present in 3D view. Our representation is designed to serve namely for comparison of multiple proteins or a set of time steps of molecular dynamics simulation.

**Conclusions:**

The novel representation is demonstrated on two usage scenarios. The first scenario aims to compare a set of proteins from the family of cytochromes P450 where the position of the secondary structures has a significant impact on the substrate channeling. The second scenario focuses on the protein flexibility when by comparing a set of time steps our representation helps to reveal the most dynamically changing parts of the protein chain.

## Background

Studying the structure of proteins has been in the scope of researchers for many decades, namely because of their importance in all living cells. Better understanding of their constitution and behavior helps to understand and control their function and properties.

**Fig. 1:**
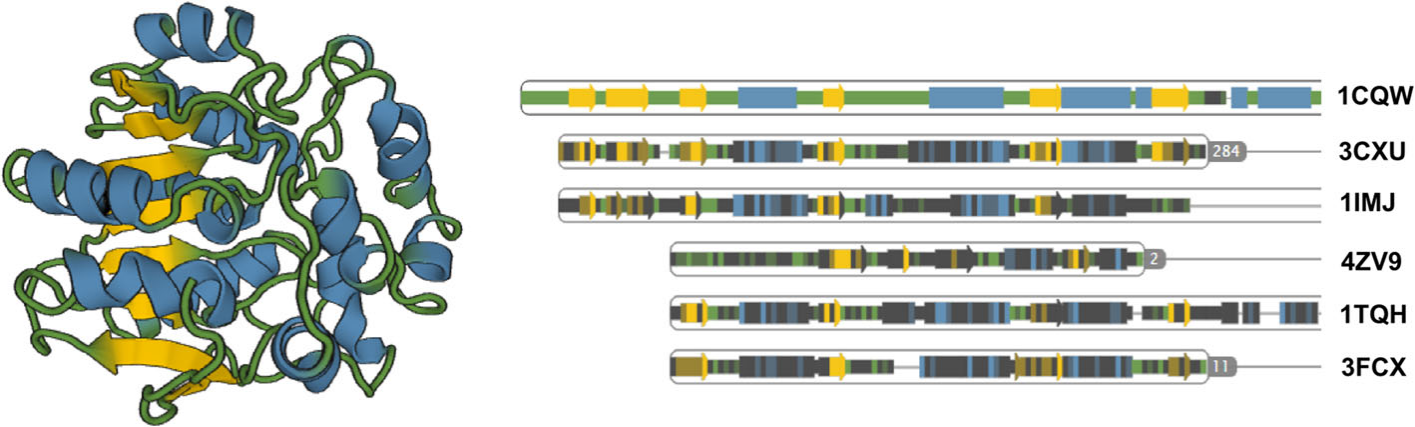
*Left* – cartoon representation of the DhaA haloalkane dehalogenase (PDB ID 1CQW). *Right* – part of the sequential representation of DhaA along with the information about secondary structures and five other proteins sequentially aligned to DhaA. The representation encodes the sequence identity of the DhaA with an automatically selected set of similar proteins. These proteins are sorted according to their similarity to DhaA. The enconding aims to convey the similarity in particular areas of the chain. Images were generated using the Aquaria tool by O’Donoghue et al. [19]

Protein structure consists of a polypeptide chain of amino acids, which is unique for each type of protein. The chain is folded into a spatial conformation that exhibits specific patterns, called secondary structures. Among these structures belong so called alpha-helices and beta-sheets. The amino acids forming these secondary structures maintain their shape thanks to weak hydrogen bonds between them. Visual representation of the protein consisting of secondary structures is denoted as cartoon or ribbon (see Fig. 1 left). This representation was first presented by Richardson in 1981 [1].

This highly abstracted visualization omits individual atoms of the protein and highlights only the protein backbone represented by the secondary structures. Such a representation is very popular among researchers because of its balanced tradeoff between the level of abstractness and conveying the spatial arrangement of the chain.

When comparing several protein structures, e.g., when searching for similar proteins in order to get the information about an unknown one, there are several existing algorithms for aligning such structures [2–6].

**Fig. 2:**
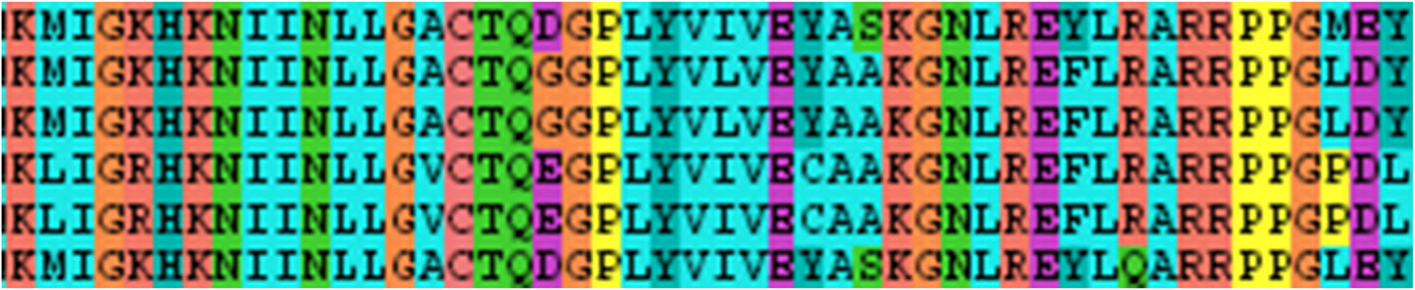
Example of the sequence alignment visualization

These algorithms align the whole structures (structure alignment) or parse the sequence of amino acids and search for corresponding patterns (sequence alignment). The results of these alignments are traditionally presented in a form of color-coded one-dimensional sequential information (see Fig. 2).

Each row represents one protein structure and the user can observe both similarities and differences between protein chains by exploring the columns. Some methods equip the sequence with the information about secondary structures (see Fig. 1 right). However, all of them lack the mutual spatial orientation of the secondary structures of the aligned proteins.

This information is crucial in many cases, namely when exploring the protein inner void space that plays a significant role in protein reactivity with other molecules. This void space is determined by the surrounding amino acids, i.e., secondary structures. Therefore, the changes in the spatial position of secondary structures directly influence the volume and shape of the void space.

**Fig. 3:**
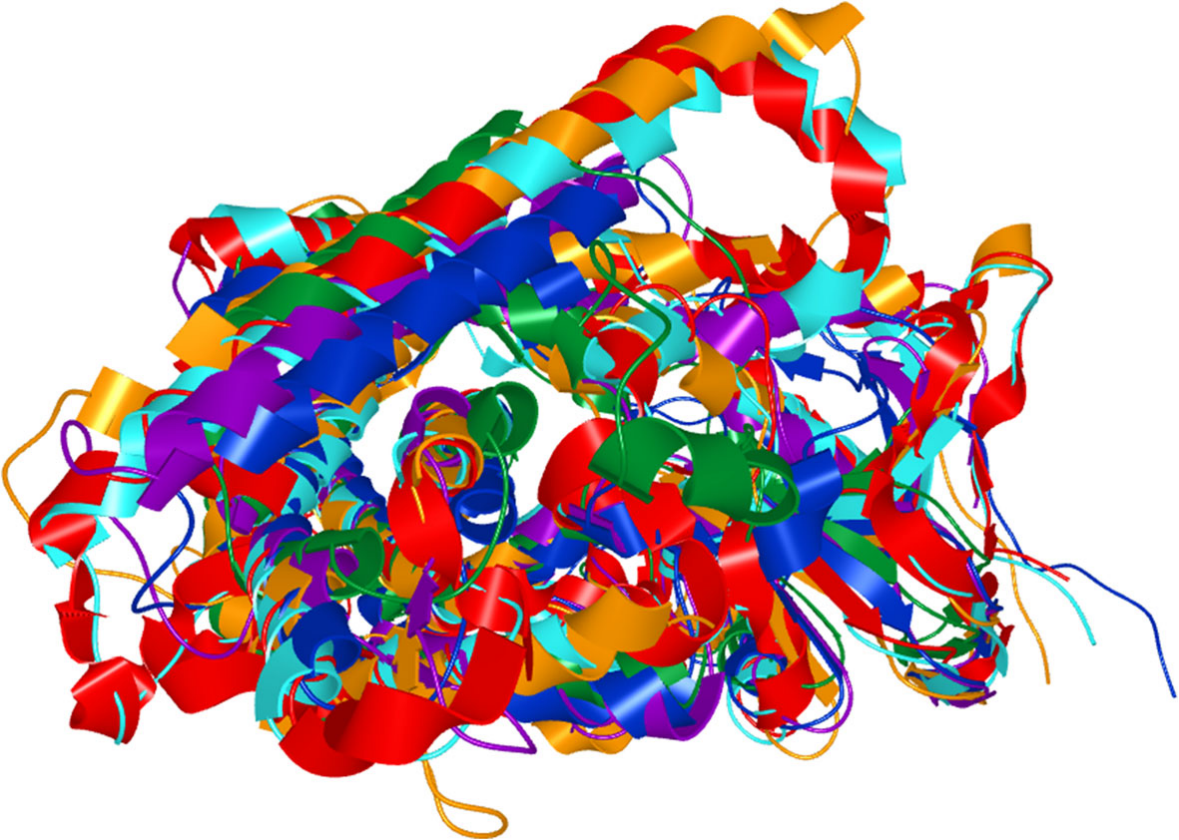
Spatial representation of structure alignment of six proteins from the cytochrome P450 family which have very similar constitution

The mutual spatial arrangement of the secondary structures can be easily observed in a 3D view. However, for comparison of multiple proteins, such a representation is very limited with respect to its scalability. In other words, due to the occlusion problems, the spatial representation is suitable for comparison of only few structures. Figure 3 demonstrates the case when six similar proteins are aligned. Even with such a small number of molecules it is hard to perceive the differences in the secondary structure positions.

To overcome the problems of the lack of mutual arrangement of the compared proteins in the sequential representation and problems with occlusion in the spatial view, we propose a new method designed to serve as a tool for comparison of multiple structures and intuitive exploration of their spatial differences. It benefits from the sequential information which consists of individual secondary structures, and when comparing this sequence with other proteins, it encodes the mutual spatial arrangement of the secondary structures of the aligned proteins. In consequence, the user can observe this arrangement without occlusion issues present in the 3D view.

In tight cooperation with the domain experts we determined the set of main requirements for the newly proposed representation. The set covers tasks which are hard to address using the existing 1D, 2D, and 3D representations. It includes the following requirements:

- It should convey the information about the constitution of the protein chain wrt. its secondary structures.
- It should serve for comparison of multiple protein chains represented by secondary structures.
- The user should be able to easily see the similarities and differences between the chains.
- The user should have the information about the global similarity between proteins.
- The user should be able to interact with the system in order to explore the similarities and differences in detail.

**Fig. 4:**
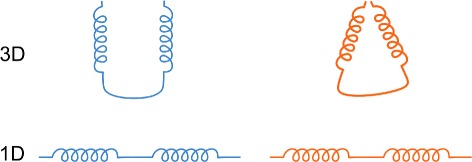
Illustration of the situation when the different spatial position determines the protein behavior. The sequential information in both situations (*blue* and *orange chains*) is the same but the spatial orientation of the helices is significantly different

These requirements reflect the need to explore the mutual position of one of the most scrutinized protein building blocks, its secondary structure. Moreover, the visual support for multiple comparison of secondary structures is currently insufficient. The existing 1D and 2D representations do not capture the mutual orientation of the secondary structures which can be critical for determining the protein properties and behavior. Such situation is demonstrated in Fig. 4 showing a simple chain consisting of two helices in two spatial arrangements. In both cases the 1D sequential information is the same. However, the spatial position is significantly different and determines the size of the entrance part (gorge) to the protein inner structure. Therefore, it can significantly influence the protein reactivity.

Some of the requirements can be addressed individually by the existing solutions but a comprehensible solution supporting all of them is still missing. Therefore, we decided to propose a solution that will cover these requirements while being simple enough and easy to use.

Our solution also utilizes the fact that the domain experts are well accustomed with the sequential representation as well as with secondary structures and their cartoon representation. Therefore, our proposed visualization is interactively linked with the 3D view. Selection of interesting secondary structures in the novel representation is directly projected to this spatial view.

### Related work

In this section we aim to cover the existing approaches to the molecule unfolding into 2D representation as well as the structural comparison of multiple molecules. In our proposed approach we aim to incorporate both these areas into a complex solution which also includes the spatial orientation of corresponding secondary structures. To the best of our knowledge, a similar tool that would address the challenge of depicting all these aspects at the same time is not yet available.

First, we review the existing approaches to abstracted 1D or 2D representation of molecules. The most typical and traditionally used technique for representation of the molecular chain is the 1D sequence of amino acids (see Fig. 2). It is present basically in all software tools for molecular visualization and are often used for the comparison of molecules and presentation of the results of alignment. The advantage is that it is easily comprehensible, but the spatial information is completely omitted.

Besides, there are many approaches aiming to unfold the molecule to a 2D view. An exhaustive and comprehensive review on the methods, tools, and applications of 2D molecular graphics was presented by Zhou and Shang [7]. The review covers numerous approaches to generation and drawing of chemical structures, protein topology representation, and schematic layout of molecular interactions. The evaluation of common visualization techniques in context of their dimensionality is covered in the work of Heinrich et al. [8]. The expected benefits and drawbacks of using manifold visualizations when solving particular tasks are discussed in order to propose ideas how to improve those techniques. Concerning the evaluation of spatial data, they claim that the 2D representation might help the user to achieve greater accuracy and lower the completion time for a given task than during the examination of 3D representation of the same data.

According to Stivala et al. [9], there are four systems specializing in automatic generation of protein structure diagrams. The main contribution of their system, Pro-origami, lies in novel approach to automatic creation of diagram layout of protein structure cartoons. This system provides diagrams which are clear, accurate, interactive, and editable. However, they are insufficient for comparison of more proteins because the representation does not preserve the similarity between them. One of the first systems was HERA [10] which generates hydrogen bonding diagrams of protein structures and optionally helical wheels and helical nets. HERA can be used for comparison of structures of proteins belonging to the same homologous family. Nevertheless, the mutual position of corresponding secondary structures is not conveyed. The TOPS cartoons, offering highly simplified description of protein topology, were the subject of system created by Westhead et al. [11]. The actual database of TOPS entries was enhanced by Michalopoulos et al. [12], enriching the topological entries with the information about packing relationships between helices and annotated them with sequence information. However, due to the simplification present in both these approaches it is impossible to understand the mutual positions of the secondary structures.

PDBsum is one of the best known atlases of summary information about each protein structure model in Protein Data Bank. A recent addition to this atlas presented by Laskowski [13] offers topology diagrams for protein domains showing the arrangement and connectivity of protein secondary structures. These diagrams are generated from hydrogen bonding plots of HERA. All above-mentioned expert systems create these simplified topology maps from atomic co-ordinates in PDB files.

An effort on providing biochemists with protein sequences supplemented with some additional information was introduced by Todd et al. in their program DOMPLOT [14]. LIGPLOT [15] program by Wallace et al. focuses on automatic generation of 2D diagrams of protein-ligand complexes as well. Another approach to creation of 2D graphs representing a protein structure is presented by Schäfer et al. [16]. In their representation, the secondary structure elements are modeled as vertices and spatial contacts between them are represented as edges. This software, also known as Visualization of Protein-Ligand Graphs (VPLG), supports several graph types and can optionally include ligand contacts.

Concerning the comparison of protein structures, Zemla presented an LGA method (Local-Global Alignment) [17] that facilitates both sequence dependent and independent modes to this problem. Other structure comparison programs use an adequate scoring function, mostly evaluating the similarity with two numbers. Those rankings are RMSD (root-mean-square deviation) between two superimposed structures together with the number of structurally aligned residues. Nevertheless, it is highly difficult to optimize both these rankings simultaneously thus they came up with a solution of many different local superimpositions that help to detect similar regions amidst the proteins. Subsequently, their scoring function has two components – it evaluates the longest continuous segments and tests global distance. Thus, this method is able to detect regions which are similar either locally or globally.

As the domain experts need to fully understand molecular mechanisms to find related structures with respect to sequence-based features, Stolte et al. integrated a visual analysis [18] in the Aquaria system [19]. The representation aims to encode the structural matches and differences of similar molecules only in the juxtaposed protein chains which can be hard to interpret. On the other hand, it allows to encode the information about individual secondary structures directly into the protein chains. This idea forms also the basis of our newly proposed visualization. The novel aspect of our approach lies in the incorporation of mutual position of corresponding secondary structures into the 2D visualization and the superposed view on selected molecules. An entirely different approach to analysis of sequences was presented by Nguyen and Ropinski [20] in their visualization technique that conveys patterns in large-scale multiple sequence alignments.

## Methods

As mentioned earlier, our approach combines the qualities of the sequential representation and 3D view and presents a novel method for comparison and interactive exploration of multiple aligned protein chains. This results in the hybrid representation which encodes the information about mutual orientation of corresponding secondary structures to the sequential view. This representation is interactively linked with the 3D view and by interactive selections the user has immediate correspondence between these two views.

**Fig. 5:**
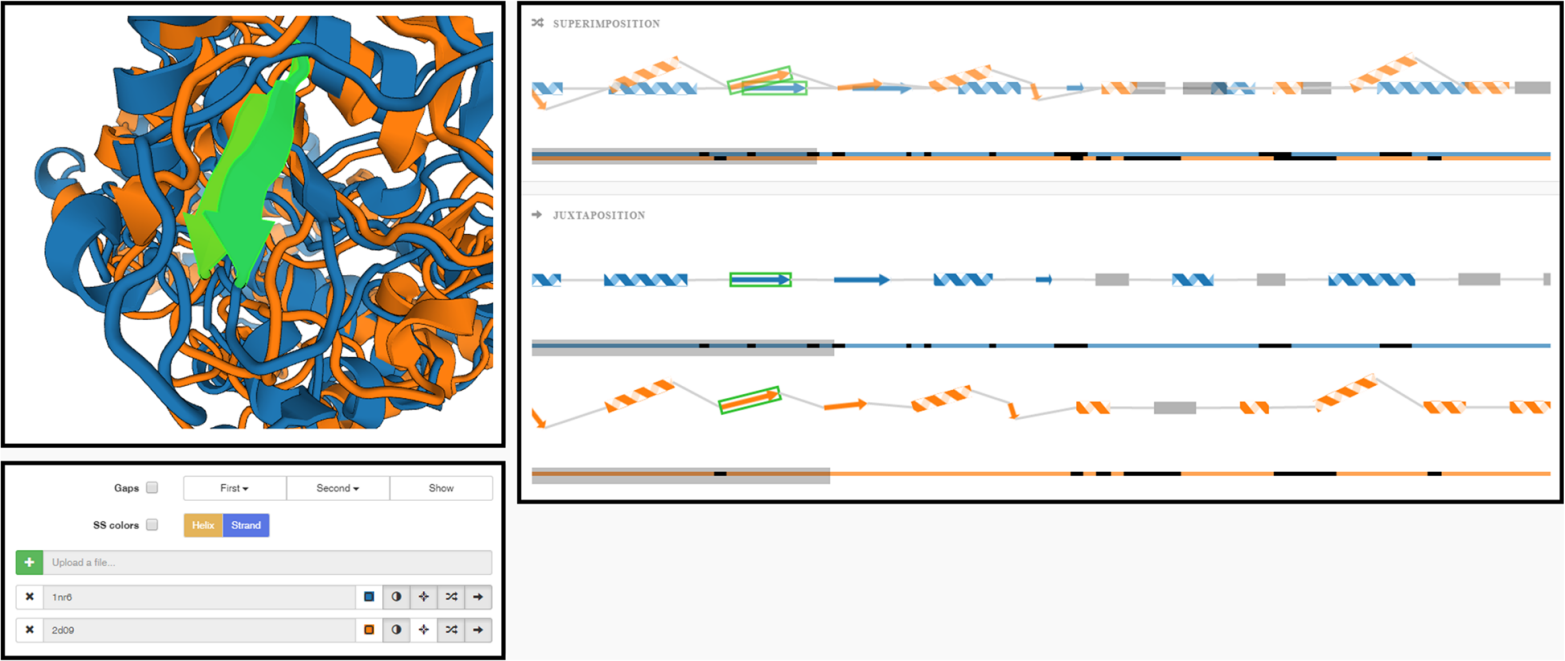
Overview of the proposed system. *Top left part* contains the 3D visualization window integrating the PV viewer [22]. The *right part* contains our proposed visualization methods serving for comparison of two or more protein chains. It consists of superimposed and juxtaposed views. *Bottom left part* contains the user interface
- Superposed and juxtaposed sequential representations of the secondary structures of aligned proteins.

The input data consists of a set of proteins in the PDB format, which are subsequently aligned with respect to their structure. The alignment is performed using the Combinatorial Extension (CE) algorithm [3]. One protein chain is selected as a reference and the remaining proteins are aligned to it. For each aligned protein this algorithm computes the transformed positions of its atoms and the RMSD number expressing the difference between the reference and the aligned protein. Furthermore, we use the DSSP algorithm by Touw et al. [21] to determine the individual secondary structures (helices, sheets) for each chain. The results are loaded to our newly proposed representation consisting of the following parts (see Fig. 5):

- 3D visualization window showing all aligned proteins (it utilizes the PV viewer available in the SWISS-MODEL tool [22]).

In the following we describe the design rationale behind the newly proposed sequential representation in detail. With respect to the requirements presented in the “Background” section we designed our 2D representation of compared protein chains, which consists of two parts – superimposition and juxtaposition panels. The superposed window aims to give the user the information about the spatial differences between the secondary structures of the aligned chains and to reveal the most significant parts (most similar or dissimilar ones). These can be further explored in detail by using the juxtaposed view and the linked 3D view. In such cases, the juxtaposition can be crucial since it does not suffer from occlusion problems. By interactive selection of desired chains in the user interface the user can explore only a selected portion of input chains.

**Fig. 6:**
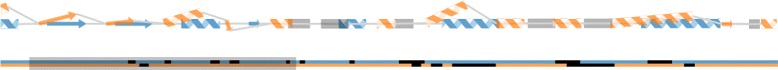
Basic element of our proposed visualization consisting of two main parts. The *top part* shows the secondary structures of the aligned proteins, the *bottom part* serves for general overview and interactive navigation and selection (*grey rectangle*)

The basic element of both superposed and juxtaposed views is depicted in Fig. 6. It demonstrates the case when two proteins chains are aligned. It consists of two main parts. The first part represents the sequential information about protein chain along with its secondary structures. Here we use three types of glyphs to distinguish between individual types of secondary structures. Arrows represent beta-sheets, spirals stand for alpha-helices, and lines represent coils. The length of the glyph corresponds to the size of the secondary structure (i.e., the number of amino acids forming the secondary structure). The reference chain is completely straightened. Then we take the information about the mutual spatial position between the secondary structures of the reference chain and the aligned chains. This determines the positioning of the glyphs representing the secondary structures of the aligned chains along the reference chain.

To be more specific, the mutual position of two glyphs representing the corresponding secondary structures is calculated in the following way. It consists of two parts, the angle and the shift. Both are derived from the mutual position of the secondary structures in 3D space. To calculate the angle between two glyphs, we take two direction vectors of the secondary structures in 3D and compute the angle between them. This value is then projected to the angle between the glyphs in 2D. To determine the shift between glyphs, we calculate the shift between the direction vectors. In our solution we simply ignore the Z coordinate but in the future we could extend this by calculating the best viewing position to minimize the skew. The length of the glyphs is taken from 3D as well by simply computing the length from the start position (first carbon atom of a secondary structure) to the end position (last carbon atom of a secondary structure).

The second part serves for interactive navigation through the protein chain in the top part. This part also gives the user the information about the relative length and global alignment of compared chains. In other words, it aims to show the mutual positioning of the aligned chains that can be of different lengths. Moreover, it enables the user to navigate through the chain and select only an interesting part of the aligned chains, which is then zoomed in the top part.

**Fig. 7:**
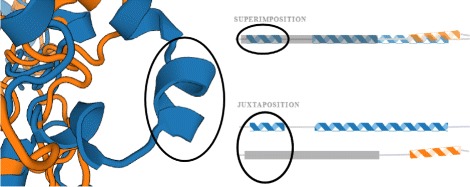
Example of a helix in the *blue* protein which does not have its couterpart in the *orange* protein. This is solved by inserting a gap (*gray rectangle*) to the superimposed and juxtaposed representations

The interactive navigation part consists of several colored lines where each line corresponds to one protein. If the protein consists of more chains, the line is interrupted. Each line might contain black parts that correspond to gaps inserted by the overlay algorithm described in the “Implementation” section. These gaps play a role of inserted parts into the straightened chains in order to maintain the correspondence between secondary structures of aligned chains. This can happen, e.g., when one chain contains a secondary structure that is missing in the second protein (see Fig. 7). In the top part each gap is represented by a gray rectangle.

**Fig. 8:**
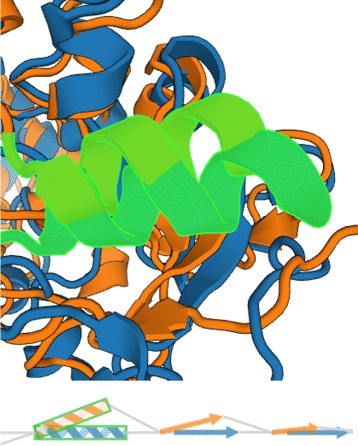
Example of encoding the mutual orientation of two corresponding helices from the aligned chains (*highlighted in green*) into our representation. Our visualization maintains the information about the “opening” of the helices

These basic elements are used in two different manners. In the first case all representations of the aligned chains are superposed so that the user can immediately see the most similar and different parts of the chains. The second case shows all aligned chains next to each other which helps to explore individual chains in detail. In both cases the user can use the navigation slider to select only and interesting part of the chains, zoom in and browse the chain in this zoomed mode. In the visualization one protein, selected as the reference one, is completely straightened. The orientation of the secondary structures in the remaining aligned chains is adjusted according to the difference between the position and rotation of the corresponding secondary structures in the reference chain (see Fig. 8).

In consequence, our abstracted representation intuitively navigates the user to the most interesting parts of the chains by linking the selection in the superposed or juxtaposed view with the 3D representation of the aligned proteins.

As mentioned before, in addition to the visualization we propose also an algorithm for solving the problem of gap insertion. This is described in detail in the following section.

### Implementation

Our system was implemented using web-based technologies in order to make it available to the wide community of potential users. Therefore, we used JavaScript along with the D3.js library [23] in order to create a fully interactive environment. Our novel visualization is linked with the 3D representation of the aligned proteins which utilizes the PV viewer [22].

In the remaining part of this section we describe in detail our proposed algorithms for solving the problems with gap insertion into our visual representation. We will outline the problem by using a metaphor when the protein chain can be taken as a thread and the secondary structures on this chain will correspond to beads put on this thread. When comparing more protein chains, we deal with a set of threads. The beads on these threads can have different colors. Their color stands for one secondary structure (helix or strand) which has its unique structure, i.e., consists of a given set of amino acids. Therefore, the beads with the same color can be positioned on different threads. In other words, if two beads being on different threads have the same color, it signifies that the corresponding secondary structures were mutually aligned and marked as the corresponding ones. Afterwards, all the threads are arranged below each other. The task is to position the beads on these threads in such a way that if they correspond to each other (have the same color), they are also positioned below each other. The beads can move along the thread but cannot exchange the position with another bead on the same thread. The following algorithm proposes a simple solution to this problem.

#### Gap insertion algorithm

The algorithm for determining the parts on the protein chains where a gap should be inserted is based on a greedy approach. The benefit lies in its simplicity and speed but thanks to its nature the output solution may not be optimal. The optimal solution would be to minimize the amount of inserted gaps. This would be very time and memory consuming since it has to process all possible solutions and select the most suitable one. Our greedy approach overcomes this and produces sufficiently correct solution in a fraction of time of the optimal solution. The sufficiency means that the number of inserted gaps does not influence the understandability of the visualization. Our solution was tested and evaluated by the biochemists who agreed that the sufficiency condition was met.

**Fig. 9:**
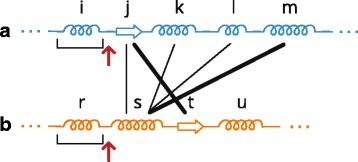
Principle of the Gap Insertion algorithm. Image illustrates the state when in proteins **a** and **b** the helices *i* and *r* were already determined as the corresponding ones and the pointers (*red arrows*) are positioned behind them. Now the strand *j* from **a** is searched in **b** and the corresponding strand *t* is found after skipping one secondary structure (*thick line*). Therefore *n*_*gap*_=1. Similarly, for helix *s* in B we search for corresponding helix in A. It is the *m* helix in A (*thick line*) and we had to skip 3 secondary structures, so *m*_*gap*_=3. So we select the first option as the next step, insert one gap to chain **a** (which will correspond to helix *s* from **b**), shift the pointers behind *j*, respectively *t*, and repeat the procedure until both proteins are processed

The algorithm operates with pairs of protein chains and it is illustrated in Fig. 9.

The idea for this algorithm comes from the double stack approach when we are able to maintain two stacks in one array. This is reached by allowing the grow of the stacks in opposite directions. The algorithm starts by positioning pointers to the beginning of both protein chains. In each step the algorithm compares the secondary structures from both chains, starting from the pointer positions. This comparison is performed in two directions, from protein A to protein B and vice versa. We will describe the principle only for one direction, from A to B. For the secondary structure at the following position from the pointer of protein A it searches for the corresponding secondary structure in protein B. The correspondence between the secondary structures is determined from their spatial distance and type. If found, it counts and remembers the number of secondary structures and their lengths (lets denote it as *n*_*gap*_) which have to be skipped in B to get to the corresponding secondary structure. The same procedure is performed for protein B, where we obtain *m*_*gap*_ as a result. Then, from these two solutions we take the one that contains less amount of skipped secondary structures. So if *n*_*gap*_<*m*_*gap*_ we insert *n*_*gap*_ gaps into protein A, just before the currently processed secondary structure. The pointer in A is set to this currently processed secondary structure, the pointer in B is shifted to the corresponding secondary structure. If *m*_*gap*_<*n*_*gap*_ we insert *m*_*gap*_ gaps into protein B and shift the pointers accordingly. If *n*_*gap*_ or *m*_*gap*_ is zero, we do not insert any gap and continue. The algorithm ends when both protein chains are processed completely. When one of the chains is already processed and the second chain still contains some remaining secondary structures, we fill the end of the processed chain with gaps.

**Fig. 10:**
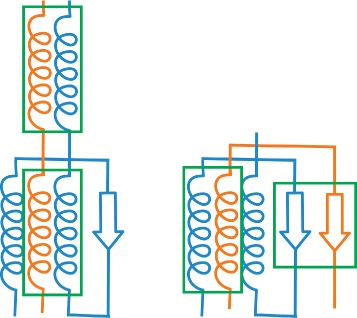
An example of the failure of the greedy approach to gap insertion. It illustrates the situation when there are more options how to select the closest secondary structures

The correctness was tested on dozens of protein structures and in several cases our greedy approach inserted a few unnecessary gaps into the chains. The algorithm can insert these unnecessary (i.e., redundant) gaps because it has no prior knowledge about the secondary structures following the currently processed position. This can happen in cases where there are more pairs of the closest structures. The situation when the greedy approach fails is illustrated in Fig. 10.

In both examples, there are two helix structures (blue) from the first protein that can be considered as the closest ones to the helix from the second molecule (orange). The subsequent secondary structures are different and crucial when considering which pair of the closest structures is correct. The correct pairs of secondary structures are highlighted in green. However, the algorithm takes the wrong pair of structures in the second example (Fig. 10 right), which results in gaps that are positioned incorrectly.

The optimal solution would create a hierarchical structure of all possible solutions and select one with the smallest number of inserted gaps. The gap insertion problem is also tightly related to the definition of the correspondence between the compared secondary structures. In other words, we need to define when two secondary structures from different chains correspond to each other. In case when the secondary structures have the same constitution, the solution is trivial. However, in many cases only a portion of the secondary structures is the same. Then it is a complex problem that has to be solved in tight cooperation with the domain experts. Their expertise should help to define a set of parameters which play a role in the similarity definition and these parameters should be incorporated into the gap insertion algorithm.

Despite this problem the domain experts concluded that these additional gaps do not decrease the readability and understandability of the visual representation.

#### Algorithm for processing molecular dynamics

**Fig. 11:**
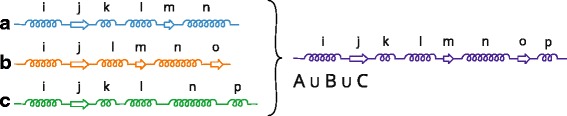
Principle of the algorithm processing molecular dynamics. Image illustrates the state when we aim to compare three time steps **A**, **B**, **C** of molecular dynamics simulation. First an artificial chain *A*∪*B*∪*C* is created as the union of secondary structures from the three input chains – time steps. Then each of these chains is compared with the artificial chain using the Gap Insertion algorithm and detected gaps are inserted into the chains

When comparing individual time steps of a molecular dynamics simulation, the situation is slightly different. We can use the fact that only small changes in its secondary structures and the constitution of the protein chain can occur over time. These changes can happen at the ends of the secondary structures where the amino acid can change its membership to the given secondary structure because of the movement of the molecule in the dynamics. If the secondary structure is very short (consists of one or two amino acids), it can completely disappear in some time steps. In this specific case we utilize a different approach, illustrated in Fig. 11.

From all time steps we derive one aggregated chain containing all secondary structures that appear at least in one time step. In this way we create an artificial chain which is internally stored and not presented to the user. This artificial chain contains one representative of each secondary structure which appeared at least in one of the time step chains. Then each time step, chain *X* is compared with this artificial chain and the necessary gaps are inserted into *X* (here we again utilize our proposed Gap Insertion algorithm). These gaps are positioned onto the places where the artificial chain contains a secondary structure but chain *X* does not possess it. When all time steps are processed, the artificial chain is removed.

### Interaction

The proposed visualization is directly linked with the 3D view. The user can interact with both views. In the 3D view the individual secondary structures are highlighted when hovering over them with mouse and the information about the type and identifier of that secondary structure appears. When selected by a mouse click, the secondary structure is highlighted in green. The 3D view can be also zoomed to be able to observe the selected parts in more detail (see Fig. 8). Similarly, the user can select individual secondary structures in the 2D view by clicking on them. When any of the secondary structures is selected, the other view highlights the element as well. This functionality gives the user an insight on the spatial positions of the selected structures. It also offers independent interaction with both views yet still in context of the selected elements.

We also provide the users with a configuration panel located below the 3D view which allows the user to load individual structures and further manipulate with them, e.g., defining the reference protein onto which the remaining proteins are aligned. Proteins in the juxtaposition view are by default sorted by the computed RMSD between the reference protein and the others. Therefore, the most similar proteins are positioned closer to the reference protein on the top. However, this order can be changed by simple interaction with the user interface. The whole interface as well as the interaction possibilities can be observed in the supplementary video.

## Results and discussion

Our proposed visualization was tested on several usage scenarios proposed by the domain experts in biochemistry, namely in protein engineering. The group of experts consisted of one professor (head of the research group), two post-doc researchers, and two PhD students. All of them are active in designing modifications of protein structures. The evaluation of our newly proposed representation was conducted in the following way. The domain experts defined the input sets of protein structures and then used the resulting representation to compare and explore them in detail. In the evaluation phase they were asked to focus mainly on the visual representation and its ability to convey the similarities and differences between the chains according to the orientation of their secondary structures. Their feedback is summarized at the end of this section.

In the following we will describe two scenarios which are the representatives of the most typical problems commonly faced by our domain experts. The first scenario shows the typical case when the spatial orientation of the secondary structures is substantial. Therefore, it is an ideal candidate for the evaluation of our proposed visualization. It aims to compare proteins from the cytochrome P450 family of proteins which are published and compared in the review paper by Cojocaru et al. [24]. The P450 cytochromes are enzymes responsible for the biotransformation of several drugs. Therefore, they play a significant role in drug design. The study presents newly revealed channels in this family of proteins. Studying these channels is of high interest because they can serve for transportation of ligands to the protein active site where a chemical reaction between the protein and ligand can occur. By studying these channels we can reveal how substrates may access the active site and how products may egress. Such channels may also be relevant for the passage of the smaller water and oxygen molecules involved in the reaction.

The study demonstrates how the changes in protein structure influence the appearance of their protein channels. Motion of specific secondary structures might cause that some channels can merge which further influences the protein properties and behavior substantially. These movements are most significant when comparing crystal structures of the mammalian and bacterial enzymes.

The authors are exploring enzymes (for our case we selected those with PDB identifiers 2D09, 1PQ2, 1IZO, 1F4T, and 1NR6) which represent different topologies of cytochrome P450. The presence and position of channels in these enzymes are distinguished by the secondary structure elements lining the channels at the protein surface. As the positioning of the secondary structures varies from one cytochrome to another, the spatial location of channels vary considerably as well. Therefore, the key to understand the differences between channels lies in the exploration of differences in the spatial positions of lining secondary structures. However, using the juxtaposed views illustrated in the paper (see Fig. 2 in [24]) or superimposing the 3D representations it is very hard to reveal the differences in positions of the secondary structures (see Fig. 3).

**Fig. 12:**
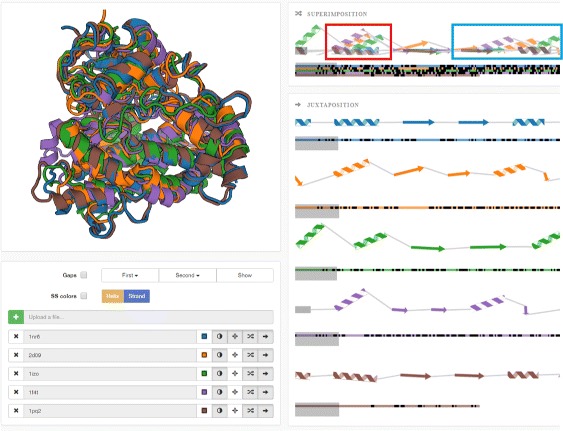
Five aligned protein structures from the cytochrome P450 family. *Top left* part shows the 3D view and *right* part represents a fraction (for better illustration) of the superimposed and juxtaposed representations of the aligned chains. The superimposed view clearly shows that secondary structures marked by *red rectangle* are well aligned and their mutual position differs only slightly. On the other hand, the secondary structures in the *blue rectangle* are highly scattered in the 3D space

Using our newly proposed representation the user can observe the differences in the aligned chains of these structures (see Fig. 12). By selecting those interesting parts in the 2D view the user is intuitively navigated to the areas in 3D space where these parts are located. This enables fast exploration of the aligned chains and changes in the void space lined by the secondary structures which determines the geometric properties of channels.

**Fig. 13:**
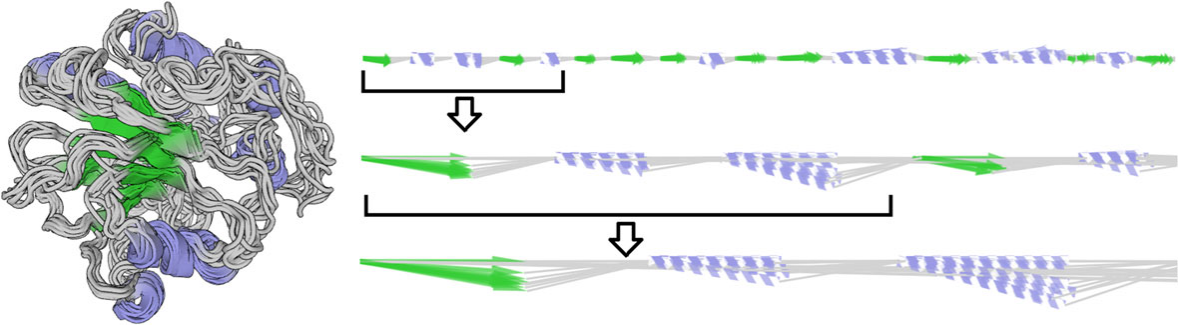
Selected subset of molecular dynamics simulation time steps in the 3D view (*left*) and the superimposed 2D representation on three different levels of detail

The second scenario focuses on the exploration of protein flexibility. This is also a hot topic in biochemistry since it was revealed that the protein function is influenced not only by its structure but also by its dynamic behavior [25]. The exploration can be reached by studying the behavior of the protein via the molecular dynamics simulations. Individual secondary structures of the protein can change their positions over time and the more significant movement, the more flexible a given secondary structure is. Therefore, we can study the protein behavior by comparing the protein chain in selected time steps. Here our representation again helps to reveal the most similar (i.e., stable) and most different (i.e., flexible) secondary structures (see Fig. 13) and to navigate the user to these parts in the 3D view.

The domain experts appreciated that our superposed view first shows the overview of the compared structures and it is easy to reveal those parts where the mutual position of the secondary structures is significantly different. These parts can be then scrutinized in more detail by easy and intuitive selecting, zooming, and highlighting. The 2D view also helped them to reveal the spatial shift between the corresponding secondary structures. They also appreciated the possibility to explore individual chains in the juxtaposed view which are sorted according to their similarity with the reference chain.

The domain experts concluded that our proposed 2D representation along with its integrated 3D view is innovative and insightful exploration system since it helps them to easily reveal the most interesting parts of the aligned protein chains. Thus, it overcomes the occlusion problems because the user is directly navigated only to the specific parts of the chains in the 3D view.

The scalability of our approach highly depends on the input data and the similarity between the scrutinized chains. Theoretically there is no limit for the number of displayed chains, the only problem can be the readability of the resulting appearance. If the differences in the constitution and spatial orientation are small the approach can be used for dozens of solutions. On the other hand, when comparing significantly different solutions, the visualization will suffer from the occlusion problems even for a very small number of chains. This can be partially suppressed by the ability to interactively select only a desired subset of proteins and thus remove, e.g., those with the most significant differences.

## Conclusions

In this paper we proposed a novel visual representation of proteins aiming to intuitively compare several aligned protein chains. The representation combines the advantages of the 2D sequential representation and the 3D view which helps to reveal the most significant parts along the chains, i.e., the similarities and differences. It helps the user to understand the mutual position of secondary structures in the aligned chains and explore them subsequently in 3D. The usability of our approach was tested on several usage scenarios and the domain experts confirmed that it helped them to reveal and understand the differences between secondary structure positions more quickly and intuitively than using the previous approaches. Among these belong namely the traditional 3D representation and different variants of the 2D unfolded representations which are mostly not suitable for comparison and lack the information about the spatial orientation of secondary structures.

The discussion with the domain experts revealed several possible extensions of our current implementation. One bottleneck of our approach currently lies in the Gap Insertion algorithm which, due to its simplicity, can insert unnecessary gaps. Therefore, we aim to design and implement more sophisticated, yet time and memory efficient, approach to insert gaps more correctly. Another possible extension, also suggested by the domain experts, is to automatically highlight interesting parts of the chains by introducing a similarity index. Naturally, this index has to be defined in tight cooperation with the biochemists.

Another bottleneck occurs when comparing many proteins or many time steps (from dozens to hundreds). Even when the compared chains are very similar, the superposed visualization becomes at some point too complex (i.e., there will be so many overlapped chains that the visualization becomes unreadable). In these cases the user is usually interested in the significant differences between the chains. Therefore, we plan to implement a contour-based visualization which will outline only the contours of the superimposed structures. Finally, our representation could be further equipped with the additional information about positions of protein channels, interacting ligands, and other biochemically relevant structures.
